# The Efficacy of Acupuncture for Treating Depression-Related Insomnia Compared with a Control Group: A Systematic Review and Meta-Analysis

**DOI:** 10.1155/2017/9614810

**Published:** 2017-02-14

**Authors:** Bo Dong, Zeqin Chen, Xuan Yin, Danting Li, Jie Ma, Ping Yin, Yan Cao, Lixing Lao, Shifen Xu

**Affiliations:** ^1^Shanghai Municipal Hospital of Traditional Chinese Medicine Shanghai, Shanghai University of TCM, Shanghai 200071, China; ^2^School of Medicine, Nanchang University, Jiangxi 330031, China; ^3^School of Chinese Medicine, The University of Hong Kong, 10 Sassoon Road, Pokfulam, Hong Kong; ^4^School of Medicine, Center for Integrative Medicine, University of Maryland, Baltimore, MD 21201, USA

## Abstract

*Objective.* To evaluate the effectiveness of acupuncture as monotherapy and as an alternative therapy in treating depression-related insomnia.* Data Source.* Seven databases were searched starting from 1946 to March 30, 2016.* Study Eligibility Criteria.* Randomized-controlled trials of adult subjects (18–75 y) who had depression-related insomnia and had received acupuncture.* Results.* 18 randomized-controlled clinical trials (RCTs) were introduced in this meta-analysis. The findings determined that the acupuncture treatment made significant improvements in PSQI score (MD = −2.37, 95% CI −3.52 to −1.21) compared with Western medicine. Acupuncture combined with Western medicine had a better effect on improving sleep quality (MD = −2.63, 95% CI −4.40 to −0.86) compared with the treatment of Western medicine alone. There was no statistical difference (MD = −2.76, 95% CI −7.65 to 2.12) between acupuncture treatment and Western medicine towards improving the HAMD score. Acupuncture combined with Western medicine (MD = −5.46, CI −8.55 to −2.38) had more effect on improving depression degree compared with the Western medicine alone.* Conclusion*. This systematic review indicates that acupuncture could be an alternative therapy to medication for treating depression-related insomnia.

## 1. Introduction

Depression is one of the most common mental disorders and is listed as the major disease causing the second-highest financial burden of diseases in China [1, 2]. Two large, recent epidemiological surveys indicated that the lifetime prevalence of depression was 16% [3, 4]. Depression has complicated influencing factors, such as loss of interest and sleep disorders [5]. Insomnia is defined as difficulty in falling asleep and in maintaining sleep (including easy to awaken, waking up too early, and sleep difficulties). It causes decreased sleep time and poor sleep quality which is affecting the ability to learn, work efficiency, and, ultimately, the quality of life. There is a complex relationship between depression and sleep disorders [6]. Insomnia and depression are often closely linked; approximately 70% of patients with depression have symptoms of insomnia [7]. The prevalence of depression in patients with insomnia is higher than that of patients without insomnia by 3 to 4 times [4], and sleep disorders are one of the primary manifestations and diagnostic criteria for depression [8]. One of the manifestations of severe depression is waking up two hours or more earlier than normal [4].

First-line antidepressant medication includes selective serotonin reuptake inhibitors (SSRIs), serotonin-norepinephrine reuptake inhibitors (SNRIs), tricyclic antidepressants (TCAS), and monoamine oxidase inhibitor (MOIs) [9]. However, these drugs are associated with a number of undesirable side effects such as weight gain, sedation, dry mouth, nausea, blurred vision, constipation, and tachycardia [9]. The effect of acupuncture treatment for depression is significant. Chan's [10] systematic review and meta-analysis of acupuncture combined with antidepressants for depression showed that acupuncture combined with antidepressants was better than the single use of antidepressants. Cheuk DKL's [11] systematic review and meta-analysis of acupuncture treatment for patients with insomnia showed that acupuncture treatment was better in improving sleep quality, decreasing sleep latency, and prolonging total sleep time, compared with the control group. The purpose of this meta-analysis was to evaluate the effectiveness of acupuncture as monotherapy and as an alternative therapy in treating depression-related insomnia.

Acupuncture is widely used in the treatment of depression-related insomnia. A large number of studies have reported that acupuncture treatment is effective in treating depression-related insomnia [12–29]. However, it has been limited by the insufficient number of high-quality, well-designed randomized controlled trials. This systematic review and meta-analysis on the acupuncture treatment for depression-related insomnia can find some results. We provide further evidence of the quantity and quality in order to draw more definitive conclusions. In this systematic review and meta-analysis, we further evaluated the effectiveness of acupuncture in the treatment of depression-related insomnia.

## 2. Methods

### 2.1. Study Inclusion and Exclusion Criteria

The flowing search terms were used: acupuncture/electroacupuncture/EA and insomnia/sleep disorders and depression. Randomized placebo or sham controlled trials of adult subjects (>18 y), who had depression-related insomnia and received acupuncture, or electroacupuncture, were included. Only papers published in English or Chinese languages were included in the study. Letters, comments, editorials, case reports, technical reports, or any nonoriginal studies were excluded. Studies presented in the outcomes of interest were not presented in a quantitative manner.

### 2.2. Strategy for Literature Search

This meta-analysis was conducted in accordance with PRISMA guidelines. Two independent reviewers (Bo Dong and Jie Ma) performed systematic searches in the following databases: (1) Chinese databases: Chinese Journal Full-text Database (CNKI); Wanfang database, Chongqing VIP database, and National Knowledge Infrastructure (NKI); (2) English databases: MEDLINE (1946–March, 2016), In-Process Citations (1950–3/2016), Publisher Supplied Citation (1950–March, 2016), OVID, EMBASE (1946–March 2016), and Cochrane Central Register of Controlled Trials (CENTRAL, January 27, 2016). We hand-retrieved the additional articles in the treatment for depression from 1979 to March 2016.

### 2.3. Data Collection and Analysis

In an independent manner, two authors (Bo D and Zq C) performed data extraction on the included trials. The data problems were solved through consultation, if necessary a third reviewer was consulted to resolve any uncertainties regarding study inclusion. The following information was extracted from studies that met the inclusion criteria: the name of the first author, year of publication, study design, demographic data of subjects, types of intervention, and outcomes. The criterion of data extraction is in accordance with the Cochrane meeting standards [30]. The quality of the data was evaluated using the Cochrane Risk of Bias Tool. We evaluated the quality of the included trials according to the following categories: Category A (good): studies have the least biases and results are considered valid. These studies consisted of clear descriptions of the study populations, the settings, and the interventions; had appropriate statistical and analytical methods; had no reporting errors; had less than 20% dropouts; had clear reporting of dropouts; and had appropriate consideration and adjustment for potential confounders. Category B (fair): studies were susceptible to bias to some degree and were not sufficient to invalidate the results. These studies may have had suboptimal adjustments for potential confounders and may have lacked certain information that was needed to assess limitations and potential problems. Category C (poor): studies had significant biases that may invalidate the results. These studies either did not consider potential confounders or did not make adjustments for them appropriately. These studies may have had critical problems in design, analysis, missing information, or discrepancies in reporting.

### 2.4. Evaluation of Bias Risk

We used the JADAD quality evaluation method to deter risk of bias in our meta-analysis studies. These are the following outcomes: (1) correct method of generating randomization numbers; (2) use of randomization; (3) implementation of blind study; (4) complete data set; (5) publication bias; and (6) possibility of other bias [8].

### 2.5. Evaluation Results

Due to the different control groups included in the 18 articles, the meta-analysis was divided into 3 parts: (1) acupuncture with Western medicine; (2) acupuncture with sham or placebo acupuncture; and (3) combination of acupuncture and medication with medication. Due to different measurement of the outcomes, not all of the articles appeared in each of the meta-analyses.

The primary outcome was the PSQI score. In 1989, Dr. Buysse, a psychiatrist at the University of Pittsburgh (US), developed the Pittsburgh Sleep Quality Index (PSQI). It has commonly been used to evaluate the quality of sleep in patients with sleep disorders and concomitant mental disorders. The secondary outcome was the HAMD (Hamilton Depression) scale. HAMD is the most widely used scale for the clinical assessment of depression. HAMD Scales (17 items, 21 items, and 24 items) are different in involved areas and total score. HAMD Scale (24 items) includes circadian variations, depersonalization, paranoid symptoms, obsessive-compulsive behaviour, feeling of decreased ability, feeling of despair, and feelings of inferiority. Some clinical trials reported the effective rate of treatment, which provides a clear description for syndromes improving degree. The definition of a clinical cure is a normal sleep time of >6 h, deep sleep, being energetic after waking up, and the reduction rate of HAMD scores being more than 75%. Effectiveness is less insomnia symptoms, increasing sleep time <3 h, and the reduction rate of HAMD being more than or equal to 25%. Invalid is no improvement after treatment or the reduction rate of HAMD <25% [31].

We used the software Revman 5.30 that is provided by the Cochrane collaboration network for data analysis. Data were summarized by using relative risk (RR) with 95% confidence intervals (CI) for binary outcomes or mean difference (MD) with 95% CI for continuous outcomes. A *χ*2-based test of homogeneity was performed and the inconsistency index (*I*^2^) and *Q* statistics were determined. If the *I*^2^ statistic was >40%, a random effects model was used. If the *I*^2^ statistic was <40%, a fixed effect model was used. Pooled effects were calculated and a 2-sided *P* value < 0.05 was considered to indicate statistical significance. The leave-one-out approach was used for sensitivity analysis of the primary outcome using. Publication bias was assessed by funnel plot.

### 2.6. Analysis of Results

**Figure 1:**
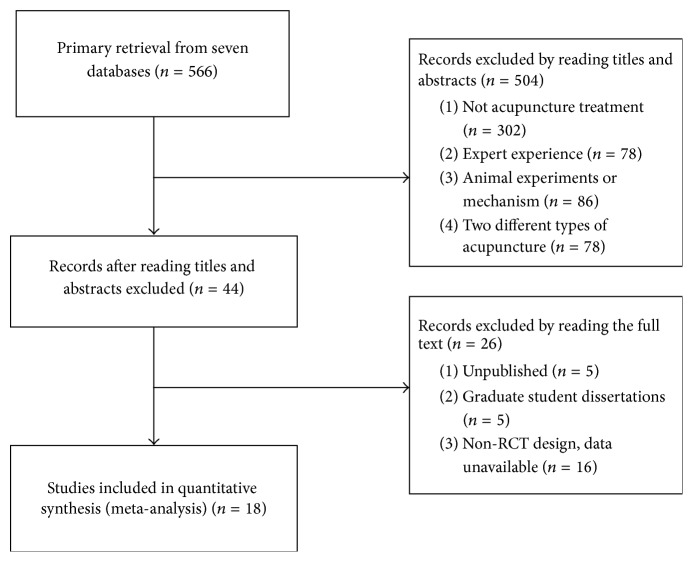
Flowchart of study selection.

**Figure 2:**
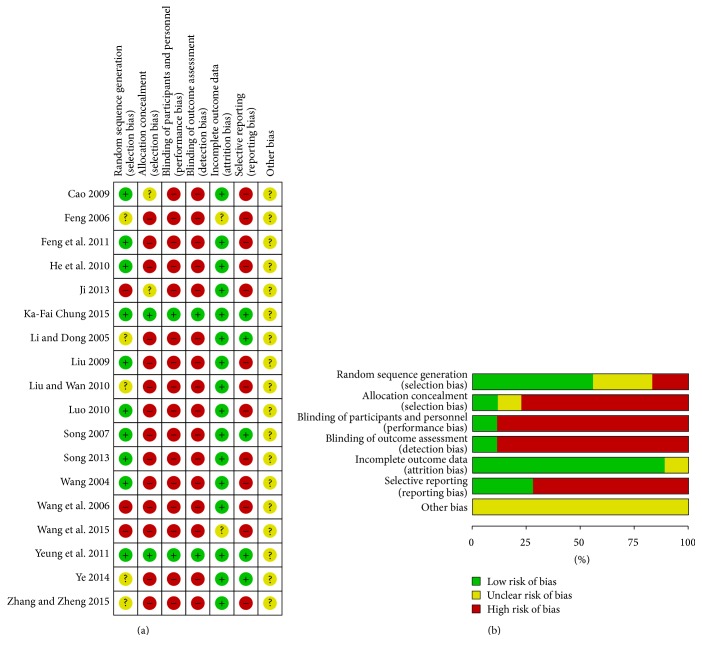
Results of quality assessment of included randomized controlled trials or prospective comparative studies. (a) Potential risk of bias of each included study. (b) Summarized risk of included studies.

In final, 566 citations were identified, and the majority was excluded for having no acupuncture treatment, case reports, letters, comments, or basic research. Data from 44 RCTs were included in this review (Figure 1). Among them, 5 trials were unpublished and 5 papers were from graduate student dissertations. Sixteen RCTs were excluded from this review due to multiple publications, non-RCT design, and unavailable data or for not having met one of the inclusion criteria. By reading full texts of the published studies, 18 studies were confirmed that they were all randomized controlled trials of acupuncture treating insomnia associated with depression (Table 1), among them 11 articles were RCTs of acupuncture compared with Western medicine; 5 were RCTs of acupuncture combined with medication compared with sole medication; 2 were studies of acupuncture compared with sham or placebo acupuncture control. Results showed that there were 1678 participants included, 908 women and 641 men ageing from 18 to 75 y old. The gender of 120 subjects remained unknown. The duration of disease ranged in length from one m to 22 y; there were 811 patients in the intervention group and 858 patients in the control group. Among the articles, 16 articles used Pittsburgh Sleep Quality Index (PSQI) [12–14, 16–29], 12 of the studies applied the Hamilton Depression Scale (HAMD) [12, 16, 18, 21, 23–29], and 5 articles used the self-rating depression scale (SDS) [13, 14, 28, 29]. Four articles observed the follow-up period and the recurrence rate [13, 14, 28, 29], and 6 articles studied the adverse reactions with treatment emergent symptom scale (TESS) [16, 17, 21, 23, 28, 29]. The quality of the data was not high. There was a high risk of detection bias due to no implementation of blind study and allocation concealment in more than half of the studies (Figure 2).

### 2.7. Acupuncture versus Western Medicine

**Figure 3:**
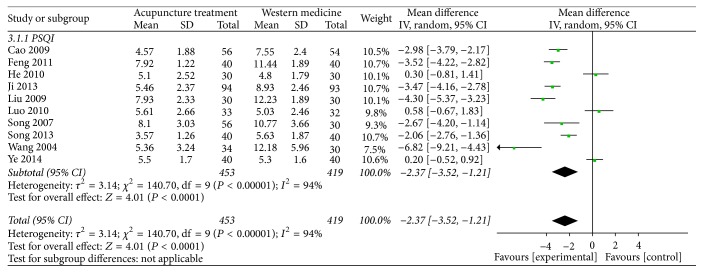
Meta-analysis for PSQI score of acupuncture versus Western medicine.* Note*. Mean: the average of the outcomes; SD: standard deviation; total: the count of the patients; weight: the credibility of the test; IV: variance methods; random: random effects model; CI: confidence interval.

A total of 10 articles reported the Pittsburgh Sleep Scale. Among them, 7 articles showed acupuncture was more effective than medication, and 3 showed no significant difference between two groups. 10 articles with high heterogeneity (heterogeneity: Chi^2^ = 140.70, df = 9 (*P* < 0.00001); *I*^2^ = 94%) and the random effects model (MD = −2.37, 95% CI −3.52 to −1.21) were used. The forest map shows the PSQI score in the acupuncture group was lower than in the medication group. It indicates that the acupuncture treatment was more effective on insomnia and depression than medication (Figure 3).

### 2.8. Acupuncture Combined with Medicine versus Single Medicine

**Figure 4:**
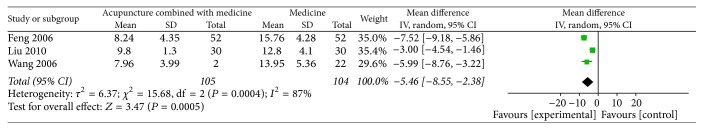
Meta-analysis for PSQI score of acupuncture combined with medicine versus single medicine.* Note*. Mean: the average of the outcomes; SD: standard deviation; total: the count of the patients; weight: the credibility of the test; IV: variance methods; random: random effects model; CI: confidence interval.

Four studies comparing the acupuncture combined with medicine and single medicine were analyzed and reported through the PSQI score, among them, 3 articles showed acupuncture combined with medication was more effective than sole medication and one showed no significant difference between two groups. The random effects model was used (MD = −2.63, 95% CI −4.40 to −0.86), due to the heterogeneity in the data (heterogeneity: Chi^2^ = 17.00, df = 3 (*P* = 0.0007); *I*^2^ = 82%). The PSQI scores in the acupuncture combined with medicine groups decreased significantly compared to the sole medication groups (Figure 4).

### 2.9. Electroacupuncture versus Sham Acupuncture or Placebo Acupuncture

**Figure 5:**
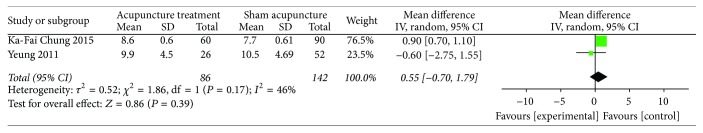
Meta-analysis for PSQI score of electroacupuncture versus sham acupuncture or placebo acupuncture.* Note*. Mean: the average of the outcomes; SD: standard deviation; total: the count of the patients; weight: the credibility of the test; IV: variance methods; random: random effects model; CI: confidence interval.

For a total of two articles based on the PSQI scores to evaluate effect, one article showed no significant difference in PSQI score between two groups and another one showed the control group was more effective than the electroacupuncture group, Heterogeneity was detected (heterogeneity: Chi^2^ = 5.72, df = 1 (*P* = 0.02); *I*^2^ = 83%), and the random effects model was used (MD = 0.17, 95% CI −1.68 to 2.03). The forest plot showed that there was no difference in PSQI score between the electroacupuncture group and the sham acupuncture group or the placebo acupuncture group (Figure 5).

### 2.10. Acupuncture versus Western Medicine

**Figure 6:**
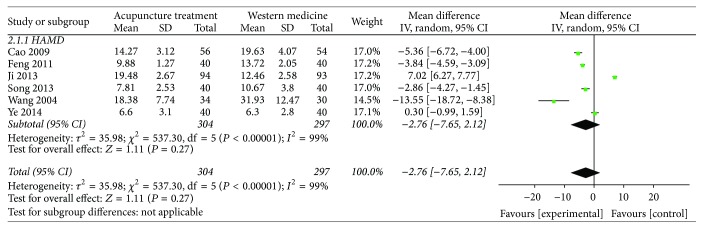
Meta-analysis of HAMD score of acupuncture versus Western medicine.* Note*. Mean: the average of the outcomes; SD: standard deviation; total: the count of the patients; weight: the credibility of the test; IV: variance methods; random: random effects model; CI: confidence interval.

A total of 6 articles reported the HAMD score. The random effects model was used (MD = −2.76, 95% CI −7.65 to 2.12); heterogeneity in the data was detected (heterogeneity: Chi^2^ = 537.30, df = 5 (*P* < 0.00001); *I*^2^ = 99%). 4 articles showed acupuncture was more effective than Western medicine, one showed no significant difference between two groups, and one showed the control group was more effective than the acupuncture group. There were no differences between the acupuncture groups and the medication groups in HAMD score. However, two medication groups showed improvement in depressive symptoms after treatment (Figure 6).

### 2.11. Electroacupuncture versus Sham Acupuncture or Placebo Acupuncture

**Figure 7:**
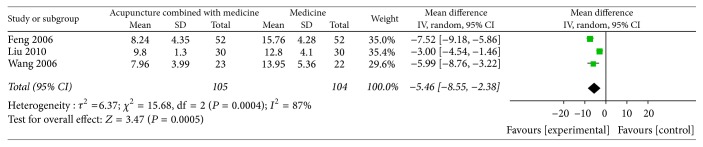
Meta-analysis of HAMD score of acupuncture combined with medicine versus single medicine.* Note*. Mean: the average of the outcomes; SD: standard deviation; total: the count of the patients; weight: the credibility of the test; IV: variance methods; random: random effects model; CI: confidence interval.

A total of two articles have a contrast between electroacupuncture and sham acupuncture or placebo acupuncture. One study showed no difference in HAMD score between two groups; another one showed the control group was more effective than the electroacupuncture group. Heterogeneity was found (heterogeneity: Chi^2^ = 1.86, df = 1 (*P* = 0.17); *I*^2^ = 46%), and we used the random effects model (MD = 0.55; 95% CI −0.7 to 1.79), (OR = 0.89, 95% CI 0.69 to 1.08). The forest plot showed that there was no difference in HAMD score between the electroacupuncture group and the sham acupuncture group or the placebo acupuncture group. The high heterogeneity may be caused by the inconsistent timing of evaluation after the treatment (Figure 7).

### 2.12. Acupuncture Combined with Medicine versus Single Medicine

**Figure 8:**
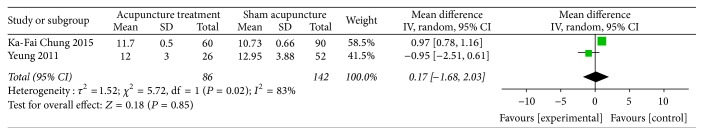
Meta-analysis of HAMD score of electroacupuncture versus sham acupuncture or placebo acupuncture.* Note*. Mean: the average of the outcomes; SD: standard deviation; total: the count of the patients; weight: the credibility of the test; IV: variance methods; random: random effects model; CI: confidence interval.

A total of 3 articles reported HAMD score. All the articles showed acupuncture combined with medication was more effective than the sole mediation. There was an obvious heterogeneity of these three articles (heterogeneity: Chi^2^ = 15.68, df = 2 (*P* = 0.0004); *I*^2^ = 87%); the random effects model (MD = −5.46, CI −8.55 to −2.38) was used. The results showed that the acupuncture combined with medicine group was more effective in improving depression degree than that in sole medication treatment group. The appearance of high heterogeneity may result from the different versions of the HAMD score and the inconsistent timing of evaluation after the treatment (Figure 8).

### 2.13. Acupuncture versus Western Medicine

**Figure 9:**
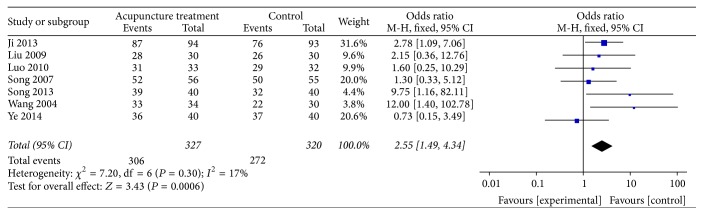
Meta-analysis of effective rate of the treatment of acupuncture versus Western medicine.* Note*. Events: the effective number of patients; total: the count of the patients; weight: the credibility of the test; M-H: Mantel-Haenszel methods; fixed: fixed effects model; CI: confidence interval.

A total of 8 articles reported the effective rate. 4 articles showed acupuncture was more effective than Western medicine group; 4 showed no significant difference between two groups. The forest plot showed that effective rate of acupuncture treatment is higher than Western medicine alone (OR = 2.34, 95% CI 1.42–3.84) (heterogeneity test: Chi^2^ = 7.81, df = 7 (*P* = 0.35); *I*^2^ = 10%) (Figure 9).

### 2.14. Publication Bias

**Figure 10:**
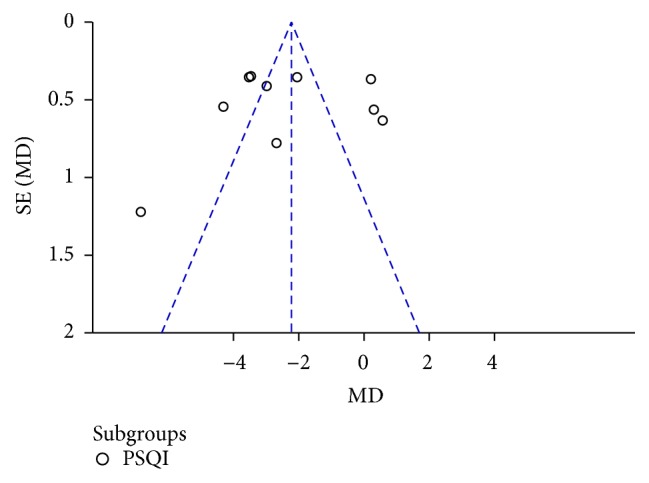
Publication bias for PSQI score of the treatment of acupuncture versus Western medicine. The funnel plot was asymmetric: four articles were on the right side of the line and 6 on the left.

Publication bias was reported via Begg's funnel plot (Figure 10), where asymmetry of the plots may have arisen through publication bias and the relationship between trial size and effect size.

## 3. Result

This systematic review and meta-analysis assessed the effectiveness of acupuncture in treating depression-related insomnia. In patients' PSQI score, after treatment, acupuncture or acupuncture combined with medication was lower than sole medication (MD = −2.37, 95% CI −3.52 to −1.21) (MD = −2.76, 95% CI −7.65 to 2.12); however, the score in acupuncture group was higher than sham or placebo acupuncture control (MD = 0.17, 95% CI −1.68 to 2.03). The effective rate of acupuncture treatment was higher than medication (OR = 2.34, 95% CI 1.42–3.84). For the HAMD score, there was no significant difference between acupuncture and medication (MD = −2.76, 95% CI −7.65 to 2.12), acupuncture combined with medicine reduced the score of HAMD than sole medication (MD = −5.46, CI 8.55 to −2.38), and there was no significant difference between acupuncture treatment and sham or placebo acupuncture control (MD = 0.55. 95% CI −0.7 to 1.79). Sensitivity analysis using the leave-one-out approach indicated the findings are robust and not dependent on any one study. Publication bias was detected.

## 4. Discussion

The object of this systematic review and meta-analysis is to evaluate the effectiveness of acupuncture in treating depression-related insomnia. We found that, compared to medication, acupuncture treatment was effective in improving depression-related insomnia and depression degree. However, compared to sham or placebo acupuncture control, there was no significant difference. This may be caused by the treatment period, the selection of points, or the level of the Acupuncturist's technical ability not being balanced.

We searched for recent systematic reviews of depression-related insomnia in English databases, but we did not retrieve any results. There was one review found in the Chinese Journal of Clinical Acupuncture in 2013 [32]. The quality of the research was relatively low, and 50% of them had been included in the thesis of graduate students. What they mainly compared is that the score of PSQI, HAMD, and the efficiency after the treatment, and the conclusion showed that the therapeutic effect of the treatment group is better than or equivalent to the controls. We made a more comprehensive search in Chinese and English databases, and 18 randomized controlled trials were included in the study. The quality of literatures included in our review is relatively higher than that in the other study. All literatures that we incorporated are randomized clinical trials and published in domestic and foreign journals, and they not only compare clinical curative effect but also assess the risk of bias evaluation and the quality of every piece of literature.

However, there were several limitations in this systematic review. The acupoints used in the included studies were heterogeneous, which may have affected the outcomes. The treatment duration of each study was different; for example, the shortest treatment period was 18 days, while the longest was 3 ms. The random methods were not clear enough and there were some errors in the random methods in some studies. Only 3 of the 18 articles were published in English. Other studies were published in Chinese. The diagnostic criteria used in the articles were not the same. Some articles used Chinese diagnostic criteria; 3 articles used the diagnostic criteria in USA. Only 3 RCTs mentioned the symptom's severity in diagnosis criteria, 1 of them reported that the participants must be mild to moderate depression, and two reported that the subjects were excluded if they had significant suicidal risk. Patients included in the studies differed in age. And there was no study reporting the calculation of sample size; 12 of 18 studies offered a detailed description of the specific steps of acupuncture treatment. In the research, there is little mention of the detailed methods used in treating the control groups. According to the funnel plot, there is publication bias, which may demonstrate that the authors had no confidence in their published trials if they resulted in a negative conclusion. The quality of the results of meta-analysis was determined by the quality of the RCT and by sufficient clinical evidence. Thus, if we want to draw a reasonable conclusion for a meta-analysis, we need larger sample sizes and more rigorously randomized controlled trials.

In conclusion, research evidence supported the use of acupuncture as an effective treatment to improve symptoms of depression-related insomnia. Compared with conventional Western medicine, acupuncture may be more effective in decreasing PSQI score. With regard to HAMD score, there is no significant difference between acupuncture and conventional Western medicine. Acupuncture combined with medicine showed to be significantly effective in decreasing score of PSQI and HAMD compared to sole medication. Current existing evidence allows limited conclusions to be reached through comparing acupuncture and medicine, and additional trials are needed to improve the reliability of these findings. In terms of adverse events, acupuncture was linked to rare and slightly adverse events such as hematomas or pain, but these resolved quickly and no other serious events have been reported. In addition, the use of acupuncture should be considered an appropriate alternative treatment for depression-related insomnia.

## Figures and Tables

**Table 1:** Summary of basic characteristics of the included studies.

First author (year)	Sample size (male/female)		Random method	Treatment	Control	Frequency	Acupoints	Place	Time	Course	Outcome measurements	Follow-Up	Safety	Assessment of bias
Cao (2009)	60 (34/26)	60 (31/29)	The random number table	AC	Estazolam	Per day	GV20, H7, EX-HN1, SP6, B62, K6	Guangdong	>1 m	Not reported	HAMD; PSQI	Not reported	Not reported	+; ?; −; +; −; +; −; ?
Feng (2011)	40 (26/14)	40 (27/13)	The random number table	AC	Fluoxetine	Per day	ST40, SP9, SP10, SP6, EX-HN3, DU20, EX-HN1, PC6, TF4	Beijing	>1 m	30 d	HAMD; PSQI; SDS	Not reported	Not reported	+; −; −; −; +; −; ?
He (2010)	30 (11/19)	30 (7/23)	The random number table	AC	Estazolam	Per day	GV20, GV17, G19, ST36, P6, H7, SP6, LIV3	Guangdong	>1 m	4 w	SAS; SDS; PSQI	Not reported	Not reported	+; −; −; −; +; −; ?
Ji (2013)	93 (39/54)	94 (41/53)	Visiting sequence	AC	Trazodone	Per day	H7, GV20, GV29, LI4, LIV3	Jiangsu	>1 m	20 d	PSQI; HAMD; ASBERG; effective rate	Not reported	Y	−; −; −; −; +; −; ?
Liu (2009)	30	30	The random number table	AC	Clonazepam	Per day	Sishenzhen, P6, SP6, Dingshenzhen, B62, K2	Guangdong	>1 m	18 d	PSQI; SDS; effective rate	1–3 m	Not reported	+; −; −; −; +; −; ?
Luo (2010)	33 (10/23)	33 (11/21)	Computer software SPSS	AC	Trazodone and Mesyre	Per day	H7, GV20, GV29, LI4, LIV3	Jiangsu	2 m~3 y	4 w	PSQI; SDS; SERS	Not reported	Y	+; −; −; −; +; +; ?
Song (2007)	60	60	Computer software	AC	Estazolam	5 times, one week	GV20, EX-HN1	Beijing	2 m~2 y	4 w	SDS; PSQI; SERS	No	Y	+; −; −; +; +; +; ?
Song (2013)	60	60	The random number table	AC	Fluoxetine	Per day	GV20, GV24, B62, K2	Hebei	1–6 m	4 w	HAMD; PSQI; effective rate; SERS	Not reported	Y	+; −; −; −; +; +; ?
Wang (2004)	34 (22/12)	30 (17/13)	The random number table	AC	Estazolam	5 times, one week	H7, P6, CV12, ST36, K3	Beijing	>1 m	4 w	HAMD; PSQI; effective rate	Not reported	Y	+; −; −; −; +; −; ?
Wang (2015)	45 (20/25)	45 (16/29)	Treatment sequence	AC	Mirtazapine	Per day	SP6, H7, EX-HN1	Jilin	>1 m	2 m	HAMD; PSQI; effective rate	Not reported	Not reported	−; −; −; −; ?; −; ?
Ye (2014)	40 (9/31)	40 (12/28)	Not reported	AC	Mirtazapine	Per day	H7, SP6, GV29, LIV3, GB34, ST36, P5	Shanghai	>3 m	3 m	HAMD; PSQI; effective rate	Not reported	Not reported	?; −; −; −; +; +; ?
Yeung (2011)	26 (6/20)	52 (10/42)	Software	EA	Sham AC and placebo AC	3 times, one week	EX-HN3, GV20, Ear Shenmen, EX-HN1	Hong Kong	>1 m	21 d	PSQI; HAMD; ISI	1 m	Y	+; +; +; +; +; +; ?
Ka-Fai Chung (2015)	60 (14/46)	90 (17/73)	Software	EA	Sham AC and placebo AC	3 times, one week	EX-HN3, GV20, and Ear Shenmen, EX-HN1, EX	Hong Kong	>1 m	21 d	PSQI; HAMD; ISI	1 m	Y	+; +; +; +; +; +; ?
Wang (2006)	23 (12/11)	22 (10/12)	Treatment date	AC combined with antidepression drugs	Antidepression drugs	Per day	GV24, GV20, GV14, DU11, CV9	Jiang Su	Not reported	4 w	HAMD; PSQI	Not reported	Not reported	−; −; −; ?; ?; −; ?
Feng (2006)	52 (38/14)	52 (34/18)	Not reported	AC combined with ultraviolet oral fluoxetine and alprazolam	Fluoxetine and alprazolam	Per day	P6, K3, H7, CV12, ST36	He Nan	>1 m	20 d	HAMD; PSQI	3 m	Not reported	?; −; −; −; −; −; ?
Li (2005)	26 (10/16)	24 (9/15)	Not reported	AC combined with fluoxetine	Fluoxetine	Per day	EX-HN22, GV20, GV29	Bei Jing	2 m~22 y	28 d	Effective rate	Not reported	Not reported	?; −; −; −; +; −; ?
Liu (2010)	30	30	Not reported	AC combined with Wuling Capsule	Wuling Capsule	Per day	GV20, GV29, H7, GV26, EX-HN1	He Nan	1~12 y	4 w	HAMD; PSQI; TESS	Not reported	Not reported	?; −; −; −; +; −; ?
Zhang (2015)	30 (18/12)	30 (23/7)	Not reported	AC combined with Guipi decoction	Mirtazapine	Per day	H7, GV29, GV20, EX-HN22, ST36, SP6, P6	Guang Dong	>1 m	4 w	SDS; PSQI	Not reported	Not reported	?; −; −; −; +; −; ?

*Notes*. AC: acupuncture; EA: electroacupuncture; SERS: rating scale for side effects; TESS: treatment emergent symptom scale; ISI: insomnia severity index. The risk of bias evaluation: random sequence generation; allocation concealment; using blind method; the blind assessment; data integrity; selective reporting bias; other forms. −: high risk, +: low risk; ?: not reported. The ratio of male to female was not reported in WH2009 Song, SC2013 Liu and Song Q2007.
